# Real-World Outcomes After Sublobar Resection for Small Node-Negative Lung Adenocarcinoma: SEER Benchmarking and a Clinicopathological Analysis

**DOI:** 10.3390/cancers18182941

**Published:** 2026-09-10

**Authors:** Yuhao Jing, Zhenbao Zhou, Miao Li, Guangzhi Sun, Xin Wang, Kai Wang, Yifan Zhao, Songxiang Li, Xiaoteng Jia, Han Zhang, Yuhang Wang, Xin Li

**Affiliations:** 1 Department of Thoracic Surgery, Tianjin Chest Hospital, School of Medicine, Tianjin University, Tianjin 300222, China; 2 Department of Thoracic Surgery, Tianjin Haihe Hospital, Tianjin University, Tianjin 300350, China; 3 Department of Thoracic Surgery, Cangzhou Hospital of Integrated Traditional Chinese and Western Medicine, Cangzhou 061000, China; 4 Department of Thoracic Surgery, Bin Hai Hospital, Peking University, Tianjin 300450, China

**Keywords:** lung adenocarcinoma, sublobar resection, lobectomy, real-world evidence, core high-risk pathological features, lymph node assessment, recurrence

## Abstract

Background: Randomized trials support sublobar resection for selected small peripheral lung cancers, but real-world outcomes may vary by procedure type, nodal assessment, and pathological risk. Methods: We performed a two-stage real-world analysis. Stage 1 included 22,864 patients with lung adenocarcinoma ≤3 cm from a node-assessed N0M0 SEER cohort. Stage 2 included 872 patients with node-negative lung adenocarcinoma from a multi-institutional, single-center-dominant clinicopathological cohort. The primary endpoint was disease-free survival (DFS). Results: In SEER, sublobar resection was associated with higher all-cause mortality (HR 1.31, 95% CI 1.23–1.38) and lung cancer-specific death (HR 1.38, 95% CI 1.27–1.51). Procedure-specific estimates were smaller for segmentectomy than for wedge resection and were attenuated after adjustment for examined lymph-node count. In the clinicopathological cohort, the primary postoperative model showed a higher DFS hazard with sublobar resection (HR 1.75, 95% CI 1.08–2.83), with a similar estimate after overlap weighting (HR 1.75, 95% CI 1.08–2.85). However, the association was not statistically clear after propensity-score matching (HR 1.41, 95% CI 0.76–2.63), 60-month restriction (HR 1.09, 95% CI 0.58–2.03), or restriction to a comparable-follow-up cohort (HR 1.57, 95% CI 0.88–2.78). The proportional-hazards assumption for DFS was violated, and late estimates were based on sparse risk sets and were unstable. The recurrence association was predominantly locoregional, and increasing Core-HRPF burden was associated with progressively worse prognosis. Conclusions: Real-world sublobar resection is a heterogeneous exposure. Observed outcome associations varied by procedure type, nodal assessment, analytical specification, and follow-up horizon and should not be interpreted as evidence that sublobar resection is intrinsically inferior to lobectomy.

## Simple Summary

Recent randomized trials have shown that sublobar resection can provide appropriate oncological outcomes for carefully selected patients with small peripheral lung cancers. Whether these results translate uniformly to routine practice remains uncertain because procedure type, patient selection, lymph-node assessment, and pathological risk vary substantially. We combined a large population-based registry cohort with a detailed clinicopathological cohort of patients with small, node-negative lung adenocarcinoma. Population-level unfavorable outcome associations were stronger for wedge resection than for segmentectomy and were attenuated after accounting for lymph-node examination. In the clinicopathological cohort, the disease-free survival association with sublobar resection varied across analytical approaches, with discordant risk-balanced estimates and no clear difference in fixed-horizon analyses. Recurrence differences were mainly locoregional and were more evident after wedge resection, while increasing adverse pathological-feature burden was associated with progressively worse prognosis. These findings support cautious interpretation of observational comparisons of surgical extent.

## 1. Introduction

Lobectomy with systematic lymph-node evaluation has long been the standard surgical treatment for early-stage non-small cell lung cancer (NSCLC) [1]. With the widespread adoption of thin-slice computed tomography (CT), an increasing number of small peripheral lung cancers are being detected, making the balance between parenchymal preservation and oncological control an important consideration in thoracic surgical practice. Two landmark randomized controlled trials (RCTs), JCOG0802/WJOG4607L and CALGB/Alliance 140503, have substantially reshaped the evidence base for surgical extent in this setting. JCOG0802/WJOG4607L showed that anatomical segmentectomy achieved superior overall survival and similar relapse-free survival compared with lobectomy in selected patients with peripheral NSCLC measuring ≤2 cm, although local relapse was more frequent after segmentectomy [2]. CALGB/Alliance 140503 subsequently demonstrated that sublobar resection was non-inferior to lobectomy for disease-free survival, with similar overall survival, among patients with peripheral NSCLC measuring ≤2 cm and intraoperatively confirmed node-negative disease [3,4]. Together, these trials support the oncological feasibility of parenchyma-sparing surgery under protocol-defined patient selection, nodal staging, and surgical quality requirements, while preserving concerns regarding local disease control.

Translating these findings into routine clinical practice presents distinct challenges. Trial participants were selected according to explicit anatomical and clinical criteria and underwent standardized intraoperative nodal assessment and protocol-defined surgical procedures. In routine practice, however, sublobar resection is performed in a substantially more heterogeneous population. Some patients undergo intentional parenchyma-sparing surgery for small or radiologically indolent tumors; whereas, others receive limited resection because of advanced age, impaired pulmonary reserve, frailty, or comorbidities. Moreover, observational studies frequently combine anatomical segmentectomy and non-anatomical wedge resection into a single “sublobar resection” category despite important differences in anatomical resection, achievable margins, hilar structure management, and lymph-node evaluation [5]. Consequently, outcome associations observed after real-world sublobar resection may vary according to both procedure type and the clinical context in which that procedure was selected.

Population-based registries provide sufficient sample size to evaluate long-term survival and procedure-specific outcome patterns but generally lack important determinants of surgical selection, including pulmonary function, performance status, frailty, comorbidities, surgical indication, and resection-margin information. Recurrence patterns and detailed pathological characteristics are also incompletely captured. Accordingly, associations observed in registry data cannot readily distinguish the influence of surgical procedure from confounding by indication, differences in nodal staging, or underlying tumor biology. These limitations may be particularly relevant to wedge resection, which in routine practice may be selected for patients who are less suitable for anatomical pulmonary resection.

Tumor size and node-negative status alone may also be insufficient to define a biologically homogeneous low-risk population. Micropapillary and solid growth patterns have been associated with increased postoperative recurrence and unfavorable survival, while visceral pleural invasion and lymphovascular invasion represent additional manifestations of invasive pathological behavior [6,7,8,9]. We therefore prespecified a four-item Core high-risk pathological feature (Core-HRPF) burden comprising visceral pleural invasion, lymphovascular invasion, micropapillary component, and solid component. Core-HRPF was designed as a parsimonious postoperative descriptor of pathological-risk burden rather than as a validated prognostic score, preoperative selection criterion, or treatment-decision tool.

Recent evidence further suggests that the oncological implications of limited resection may vary according to pathological risk. Yang et al. reported that, among patients with node-negative invasive lung adenocarcinoma measuring ≤2 cm, recurrence outcomes after different extents of pulmonary resection differed according to pathological-risk characteristics, with less favorable outcomes observed for higher-risk tumors treated with lesser resection [10]. These findings provide a rationale for considering postoperative pathological heterogeneity when interpreting real-world outcome associations after different surgical procedures.

Against this background, we conducted a two-stage complementary real-world analysis that separated population-level outcome associations from detailed clinicopathological interpretation. The study was not designed to re-test the non-inferiority questions addressed by randomized trials or to use observational data to refute their findings. Stage 1 used a node-assessed N0M0 cohort from the Surveillance, Epidemiology, and End Results (SEER) database to evaluate overall and lung cancer-specific survival associations and to examine procedure-specific heterogeneity and the influence of lymph-node examination. Stage 2 used a multi-institutional, single-center-dominant clinicopathological cohort with detailed pathological, nodal-assessment, survival, and recurrence information. Specifically, we examined (i) whether real-world outcome associations differed between segmentectomy and wedge resection; (ii) whether postoperative risk-standardized survival and recurrence associations were robust across alternative analytical specifications, follow-up horizons, and nodal-assessment conditions; and (iii) whether Core-HRPF burden provided graded postoperative prognostic stratification.

## 2. Methods

### 2.1. Study Design and Overall Analytical Framework

This study employed a two-stage complementary real-world analytical framework combining a population-based registry cohort with a multi-institutional, single-center-dominant clinicopathological cohort. Stage 1 served as the SEER population benchmark and evaluated associations between surgical extent and overall survival (OS) and lung cancer-specific death, with anatomical segmentectomy and non-anatomical wedge resection examined separately. Given the absence of detailed information on surgical indication, pulmonary function, comorbidity, resection margins, recurrence patterns, and several pathological characteristics in SEER, this stage was intended to characterize population-level outcome associations rather than estimate causal treatment effects.

Stage 2 used a complete-case clinicopathological cohort to characterize treatment selection, disease-free survival (DFS), OS, recurrence patterns, lymph-node assessment, and postoperative pathological risk. The two data sources therefore served complementary rather than discovery-validation roles. The study was reported in accordance with the Strengthening the Reporting of Observational Studies in Epidemiology (STROBE) guidelines [11].

### 2.2. SEER Data Source and Study Population

Case-level data were extracted from the Incidence—SEER Research Data (17 Registries, 2000–2023), released in April 2026, using SEER*Stat software version 9.0.43.0. The present analysis was restricted to patients diagnosed between 2010 and 2021.

Patients were included if they met the following criteria: (1) primary malignancy of the lung or bronchus; (2) histologically confirmed lung adenocarcinoma; (3) the study tumor was the first or only primary malignancy; (4) maximum tumor diameter of 1–30 mm; (5) registered stage IA, IB, or T1/T2a N0M0 disease; (6) receipt of wedge resection, segmentectomy, or lobectomy; (7) documentation of at least one regional lymph node examined with zero positive nodes; and (8) valid survival time and cause-specific death classification. Patients undergoing pneumonectomy, extended pulmonary resection, or unspecified procedures were excluded. Because SEER does not reliably distinguish clinically recorded N0 from pathologically confirmed pN0, the study population was defined as a strict node-assessed N0M0 (“pN0-like”) cohort requiring explicit documentation of negative regional lymph node assessment. Tumor size and TNM staging were harmonized across the AJCC 7th and 8th editions [12,13].

### 2.3. SEER Exposure, Covariates, and Outcome Definitions

The primary exposure was surgical extent, categorized as lobectomy versus sublobar resection, with segmentectomy and wedge resection combined in the latter group. To evaluate procedure-specific heterogeneity, surgical extent was additionally modeled as a three-category variable comprising lobectomy, segmentectomy, and wedge resection, with lobectomy as the reference category.

Prespecified adjustment covariates included age (modeled per 5-year increase), sex, race, diagnosis year, maximum tumor diameter, T category, tumor grade, and tumor location by pulmonary lobe. The primary outcome was OS. Lung cancer-specific death was evaluated as a secondary outcome using both cause-specific and competing-risk approaches [14].

### 2.4. SEER Statistical Analysis

Continuous and categorical baseline characteristics were summarized according to surgical procedure. Between-group imbalance was evaluated using standardized mean differences (SMDs), with an absolute SMD < 0.10 considered indicative of acceptable balance. Baseline distributions were assessed for both the binary comparison of lobectomy versus sublobar resection and the three-procedure comparison of lobectomy, segmentectomy, and wedge resection.

OS was estimated using the Kaplan–Meier method and compared using the log-rank test. Lung cancer-specific death was summarized using cumulative incidence functions, with death from other causes treated as a competing event, and compared using Gray’s test. Multivariable Cox proportional-hazards models were used for OS and cause-specific lung cancer mortality; whereas, Fine–Gray regression was used to estimate subdistribution hazard ratios for lung cancer-specific death [14].

To evaluate robustness to measured baseline differences, propensity-score overlap weighting, targeting the average treatment effect in the overlap population (ATO), and up-to-1:2 propensity-score matching, targeting the average treatment effect among the treated (ATT), were applied [15,16]. Propensity scores were estimated using the prespecified SEER adjustment covariates.

Because the extent of lymph-node examination differed substantially across surgical procedures and could contribute to residual staging differences, a prespecified sensitivity analysis was restricted to patients with an exact recorded number of examined regional lymph nodes. The number of examined lymph nodes was then added to the multivariable models. Within this identical subset, a same-cohort reference model was first fitted using the original adjustment covariates without examined-node count. Examined-node count was then added to the model to evaluate attenuation associated with additional nodal-count adjustment while holding the analysis population constant. This analysis was performed for both the binary sublobar-versus-lobectomy comparison and the three-category procedure model, allowing separate estimation of segmentectomy-versus-lobectomy and wedge-resection-versus-lobectomy associations after additional adjustment for examined lymph-node count.

### 2.5. Clinicopathological Cohort Design and Patient Selection

Stage 2 was a retrospective, multi-institutional, single-center-dominant clinicopathological cohort. Consecutive patients with primary lung adenocarcinoma who underwent curative-intent pulmonary resection between January 2012 and December 2021 were screened at four participating institutions: Tianjin Chest Hospital, Tianjin Haihe Hospital, Cangzhou Hospital of Integrated Traditional Chinese and Western Medicine, and Bin Hai Hospital, Peking University.

Patients were eligible if they were aged ≥18 years, had a maximum tumor diameter ≤ 30 mm, had clinically or pathologically node-negative (N0) and nonmetastatic (M0) lung adenocarcinoma based on available clinical and pathological staging information, and underwent lobectomy, segmentectomy, or wedge resection. Exclusion criteria included non-adenocarcinoma histology, multiple primary lung cancers, positive lymph nodes, surgical procedures outside the three prespecified categories, incomplete pathological-risk information required for the principal analysis, or missing/invalid survival information. The resulting cohort was analyzed using a complete-case approach.

Tianjin Chest Hospital served as the lead institution and contributed the majority of included patients. Patients were followed until death, the last documented clinical contact, or the administrative cutoff date of 30 June 2026.

The study was reviewed and approved by the Ethics Review Committee of Tianjin Chest Hospital (Approval No. 2025KY-018-01; 18 April 2025), and the requirement for individual informed consent was waived because of the retrospective study design.

### 2.6. Surgical Procedure and Lymph Node Assessment

Surgical procedure was classified as lobectomy, anatomical segmentectomy, or non-anatomical wedge resection. Hilar and mediastinal lymph-node assessment was recorded separately as a surgical-quality characteristic. The extent of nodal evaluation was characterized by the number of lymph-node stations examined, the total number of lymph nodes examined, and whether systematic lymph-node dissection (LND) was performed. Any documented lymph-node assessment was defined as at least one recorded examined lymph node, at least one recorded examined nodal station, or documentation of lymph-node sampling or systematic LND. Patients without such structured nodal-assessment documentation remained eligible if clinical staging supported N0 disease. To evaluate the robustness of survival associations to nodal-assessment quality, sensitivity analyses were performed after restriction to patients with systematic LND, ≥3 lymph-node stations examined, ≥6 lymph nodes examined, or both ≥3 stations and ≥6 nodes. Additional models incorporated lymph-node station count, lymph-node count, both measures simultaneously, systematic LND, or a restricted cubic spline for lymph-node count [17,18,19].

### 2.7. Core High-Risk Pathological Feature (Core-HRPF) Burden

The prespecified Core-HRPF burden comprised four postoperative pathological features: visceral pleural invasion (VPI; PL1 or PL2), lymphovascular invasion (LVI), a micropapillary component (>1%), and any documented solid component. These features were assessed by the pathology department of each participating institution according to routine histopathological practice and standardized diagnostic criteria [6,20]. No central pathology review was performed.

Core-HRPF was constructed as an unweighted burden count, with one point assigned for the presence of each component. For analysis, patients were categorized as having a burden of 0, 1, or ≥2 features. Equal weighting was selected a priori for simplicity and interpretability and to avoid data-driven weighting within the same cohort; it was not intended to imply equivalent biological or prognostic effects of the four components. Core-HRPF was used exclusively as a postoperative pathological-risk descriptor and was not considered a validated prognostic score, preoperative treatment-selection criterion, or predictive biomarker.

### 2.8. Survival, and Recurrence Outcomes

DFS was defined as the interval from surgery to the first occurrence of locoregional recurrence, distant metastasis, or death from any cause. OS was defined as the interval from surgery to death from any cause. Patients without an event were censored at the last documented clinical contact.

Recurrence was classified anatomically as locoregional or distant. Locoregional recurrence included recurrence in the ipsilateral lung, ipsilateral hilar or mediastinal lymph nodes, or pleura; whereas, distant recurrence included extrathoracic metastasis or contralateral pulmonary recurrence. Any recurrence was defined by the first documented recurrence event irrespective of anatomical pattern.

For site-specific analyses, locoregional and distant recurrence involvement were analyzed separately. For 19 patients, both locoregional and distant recurrence were recorded; because site-specific recurrence dates were unavailable, whether these components were synchronous or sequential could not be determined. These patients contributed target events to both site-specific analyses, and the two recurrence categories were therefore not mutually exclusive. For 17 patients, the recurrence site could not be classified from the available records. Alternative event-coding definitions for dual-site and unclassified-site recurrences were examined in sensitivity analyses.

### 2.9. Clinicopathological Cohort Statistical Analysis

Continuous variables were summarized as medians with interquartile ranges and categorical variables as counts and percentages. Between-group differences were assessed using the Wilcoxon rank-sum test for continuous variables and the chi-square or Fisher exact test for categorical variables, as appropriate. Standardized mean differences were used to quantify baseline imbalance. Survival functions were estimated using the Kaplan–Meier method. Cox proportional-hazards models were used to evaluate associations between surgical extent and DFS and OS. Sequential models included an unadjusted model; a clinicopathological model adjusted for age, sex, maximum tumor diameter, and Core-HRPF burden; the primary postoperative model additionally adjusted for surgery year; and a stage-based sensitivity model in which pathological stage replaced Core-HRPF burden. Hazard ratios (HRs) are reported with 95% confidence intervals (CIs).

Because Core-HRPF is available only after resection, models containing Core-HRPF were interpreted as postoperative prognostic standardization rather than as models of preoperative treatment assignment or causal treatment effects. Propensity scores were estimated using age, sex, tumor size, a quadratic term for tumor size, Core-HRPF burden, surgery year, and a quadratic term for surgery year. As complementary risk-balancing analyses, propensity-score overlap weighting targeting the average treatment effect in the overlap population (ATO) and up-to-1:2 propensity-score matching targeting the average treatment effect among the treated (ATT) were performed.

Potential follow-up was estimated using the reverse Kaplan–Meier method. To address differences in surgery year and follow-up opportunity, a comparable-follow-up sensitivity cohort restricted to patients treated during 2018–2021 was analyzed. The proportional-hazards assumption was assessed using Schoenfeld residuals. When non-proportionality was detected, complementary analyses included Cox models restricted to 60 months, time-stratified Cox analyses, and 60-month restricted mean survival time (RMST) analyses [21,22,23,24,25]. An exploratory 60-month landmark analysis was also performed to characterize the late follow-up period.

Procedure-specific analyses modeled lobectomy, segmentectomy, and wedge resection separately, with lobectomy as the reference. Recurrence outcomes were evaluated using cause-specific Cox and Fine–Gray competing-risk models adjusted for age, sex, maximum tumor diameter, Core-HRPF burden, and surgery year [14]. Sensitivity analyses further examined lymph-node assessment through restriction and additional adjustment for lymph-node station count, examined-node count, and systematic LND. The Core-HRPF analysis was additionally repeated with its four individual components entered separately. Procedure-specific, landmark, interaction, recurrence-pattern, and other sensitivity analyses were considered exploratory; no adjustment for multiple comparisons was applied. All tests were two-sided with *p* < 0.05, and analyses were conducted using R version 4.4.3.

## 3. Results

### 3.1. SEER Cohort Characteristics and Population-Level Surgical Outcomes

**Figure 1:**
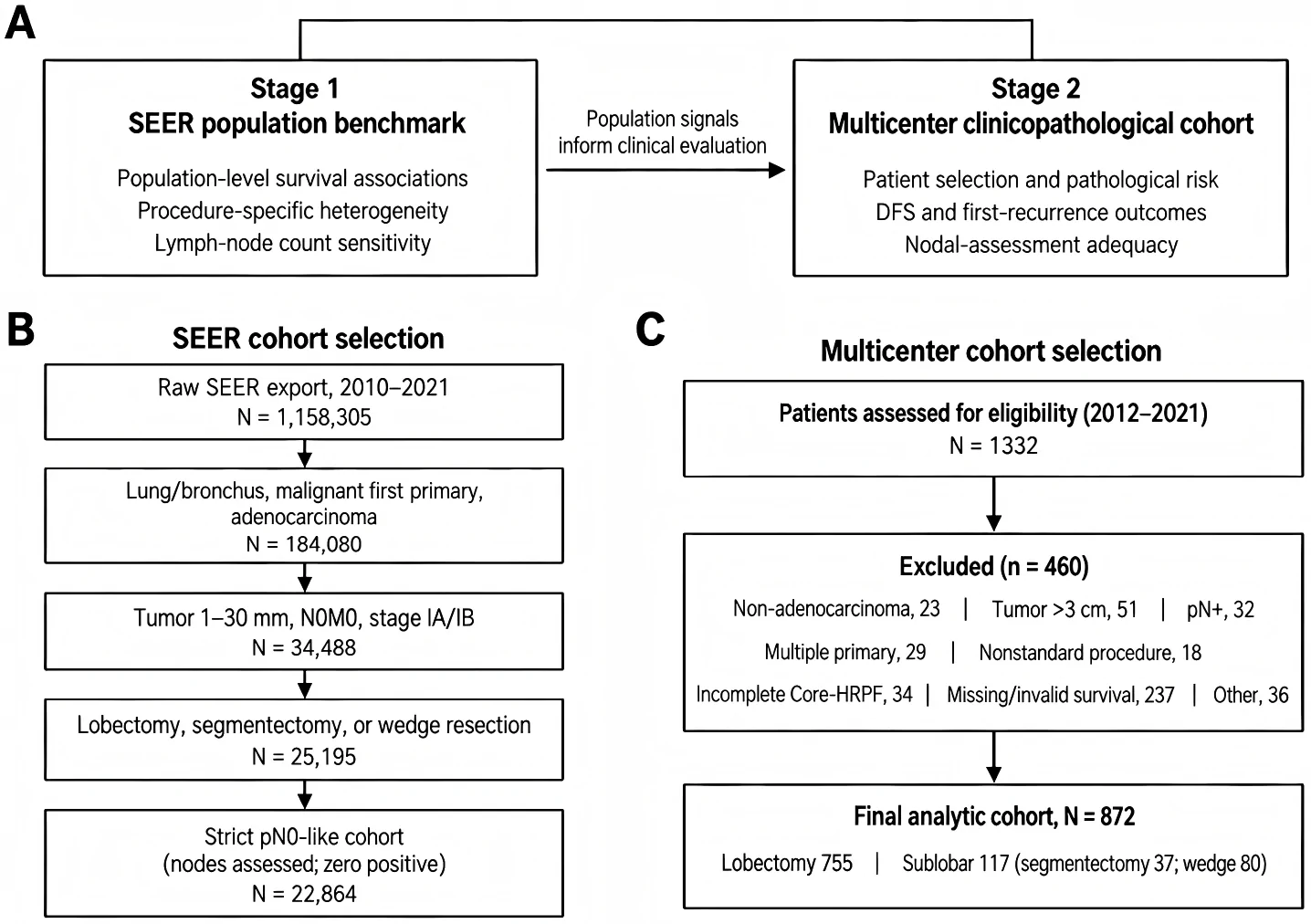
Two-stage complementary real-world analytical framework and cohort selection. (**A**) Overview of the two-stage study design. Stage 1 comprised the SEER strict node-assessed N0M0 (“pN0-like”) cohort (N = 22,864) and evaluated OS and lung cancer-specific death. Stage 2 comprised a multi-institutional, single-center-dominant clinicopathological cohort (N = 872) and evaluated DFS, OS, recurrence patterns, nodal assessment, and Core-HRPF burden. (**B**) SEER cohort-selection flow from 1,158,305 initial records to 22,864 patients in the final analytical cohort. (**C**) Clinicopathological cohort-selection flow from 1332 screened patients to 872 patients in the final complete-case cohort, including 755 lobectomies, 37 segmentectomies, and 80 wedge resections. Abbreviations: Core-HRPF, Core high-risk pathological feature; DFS, disease-free survival; OS, overall survival; SEER, Surveillance, Epidemiology, and End Results.

According to the prespecified eligibility criteria, the SEER strict node-assessed N0M0 (“pN0-like”) cohort included 22,864 patients with lung adenocarcinoma measuring ≤3 cm: 18,297 underwent lobectomy, 1556 segmentectomy, and 3011 wedge resection. Baseline characteristics according to binary and three-category surgical extent are summarized in Supplementary Table S2. During follow-up, 6920 all-cause deaths occurred, including 3333 lung cancer-specific deaths. The overall analytical framework and cohort-selection process are shown in Figure 1.

**Figure 2:**
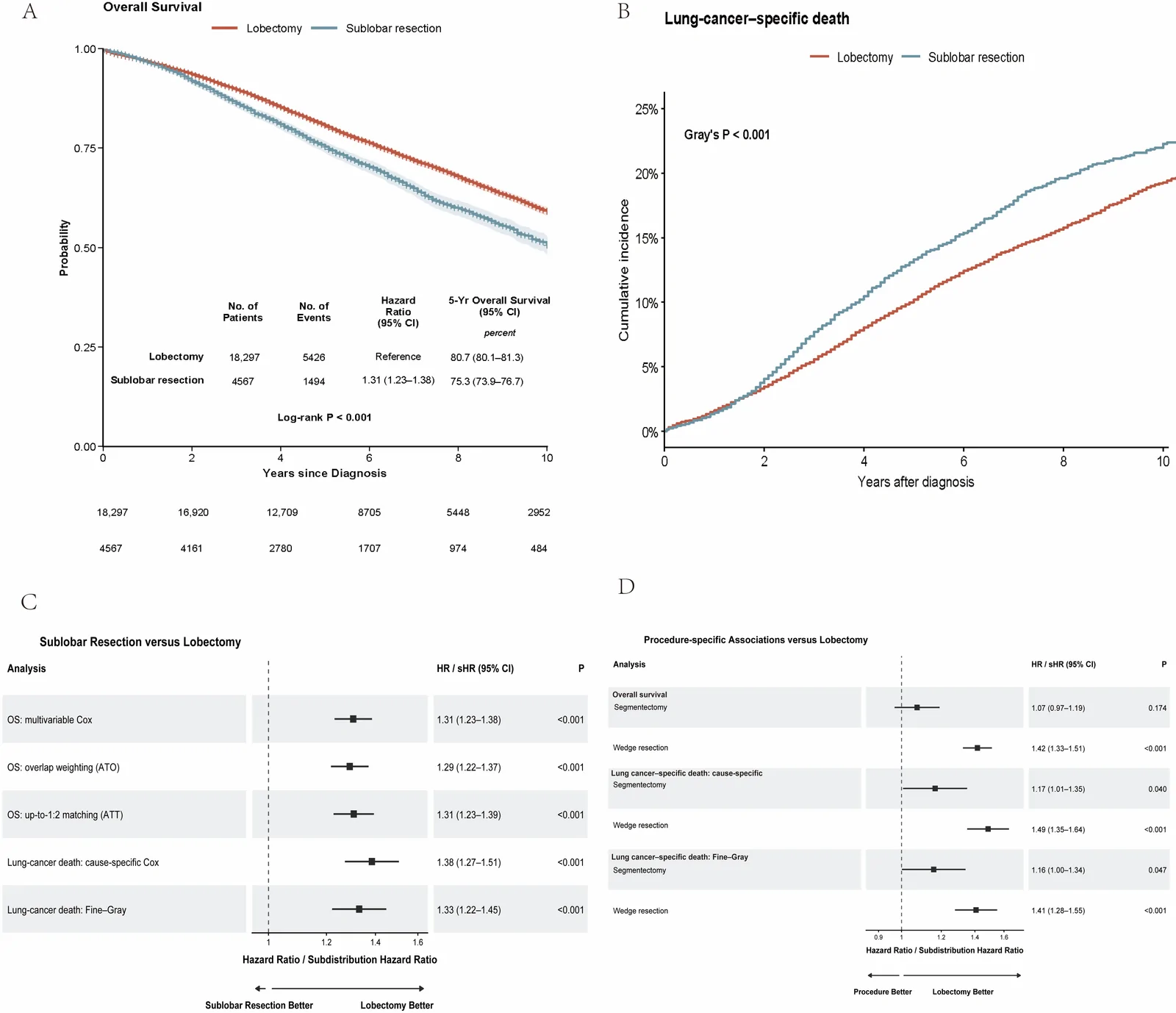
Population-level survival and procedure-specific outcome associations in the SEER cohort. (**A**) Kaplan–Meier OS curves comparing lobectomy with sublobar resection, displayed through 10 years; the log-rank *p* value and numbers at risk are shown. (**B**) Cumulative incidence functions for lung cancer-specific death, with death from other causes treated as a competing event; groups were compared using Gray’s test. (**C**) Forest plot of the binary sublobar-resection-versus-lobectomy comparison, including multivariable Cox, overlap-weighted, propensity-score-matched, cause-specific Cox, and Fine–Gray estimates. (**D**) Procedure-specific multivariable estimates for segmentectomy and wedge resection, each compared with lobectomy. HRs or sHRs >1 indicate a higher hazard associated with the index procedure; whereas, values < 1 indicate a lower hazard. Procedure-specific *p* values are nominal and were not adjusted for multiple comparisons. Abbreviations: ATT, average treatment effect among the treated; ATO, average treatment effect in the overlap population; CI, confidence interval; HR, hazard ratio; OS, overall survival; sHR, subdistribution hazard ratio.

In the binary analysis combining segmentectomy and wedge resection as sublobar resection, sublobar resection was associated with higher all-cause mortality than lobectomy in the multivariable model (HR 1.31, 95% CI 1.23–1.38; *p* < 0.001). Lung cancer-specific death showed directionally consistent associations in both the cause-specific model (HR 1.38, 95% CI 1.27–1.51; *p* < 0.001) and the Fine–Gray model (sHR 1.33, 95% CI 1.22–1.45; *p* < 0.001). Estimates after overlap weighting and propensity-score matching were similar in direction and magnitude. Five-year OS was 80.7% after lobectomy and 75.3% after sublobar resection, and the corresponding five-year cumulative incidences of lung cancer-specific death were 10.2% and 13.3%, respectively (Table 1; Figure 2).

### 3.2. Procedure-Specific Analyses and Lymph Node Examination

Procedure-specific analyses demonstrated substantial heterogeneity between segmentectomy and wedge resection. Compared with lobectomy, segmentectomy showed no statistically clear association with all-cause mortality (HR 1.07, 95% CI 0.97–1.19; *p* = 0.174). Lung cancer-specific estimates were modestly above unity, with a cause-specific HR of 1.17 (95% CI 1.01–1.35; *p* = 0.040) and a Fine–Gray sHR of 1.16 (95% CI 1.00–1.34; *p* = 0.047). Because these procedure-specific analyses were exploratory and no multiplicity adjustment was applied, the lung cancer-specific estimates should be interpreted cautiously (Table 1; Figure 2D).

In contrast, wedge resection showed larger and more consistent unfavorable associations with all three outcomes: the HR was 1.42 (95% CI 1.33–1.51; *p* < 0.001) for all-cause mortality, 1.49 (95% CI 1.35–1.64; *p* < 0.001) for cause-specific lung cancer death, and the Fine–Gray sHR was 1.41 (95% CI 1.28–1.55; *p* < 0.001) (Table 1; Figure 2D).

Within the same subset of 21,649 patients with an exact recorded examined-node count, the adjusted HRs for sublobar resection versus lobectomy were 1.29 (95% CI 1.21–1.37; *p* < 0.001) for OS and 1.39 (95% CI 1.27–1.51; *p* < 0.001) for lung cancer-specific death before adding examined-node count. After additional adjustment for examined-node count, the corresponding estimates were attenuated to 1.19 (95% CI 1.11–1.26; *p* < 0.001) and 1.26 (95% CI 1.15–1.38; *p* < 0.001), respectively. In procedure-specific models with examined-node-count adjustment, the HRs were 1.02 (95% CI 0.91–1.13; *p* = 0.762) for OS and 1.12 (95% CI 0.96–1.30; *p* = 0.160) for lung cancer-specific death after segmentectomy, and 1.27 (95% CI 1.18–1.36; *p* < 0.001) and 1.33 (95% CI 1.20–1.48; *p* < 0.001), respectively, after wedge resection. These findings indicate attenuation associated with additional nodal-count adjustment, but do not establish that nodal assessment causally explains the observed outcome differences (Table 1).

### 3.3. Patient Selection and Baseline Characteristics in the Clinicopathological Cohort

Of 1332 screened patients, 460 were excluded according to the prespecified criteria, leaving 872 patients in the clinicopathological cohort. Among these, 755 underwent lobectomy and 117 underwent sublobar resection, including 37 segmentectomies and 80 wedge resections. The cohort was strongly dominated by the lead institution, which contributed 805 of 872 patients (92.3%); the remaining three institutions contributed 42, 13, and 12 patients, respectively (Table 2; Figure 1).

Baseline characteristics differed substantially between surgical groups. Patients undergoing sublobar resection were older (median 64 vs. 61 years) and underwent surgery more recently, but had smaller tumors (median 1.1 vs. 1.8 cm; absolute SMD = 0.957), a higher proportion of pathological stage IA disease (84.6% vs. 73.2%), and a lower Core-HRPF burden (burden 0: 76.1% vs. 40.9%; absolute SMD = 0.763) than patients undergoing lobectomy. Individual adverse pathological features, including VPI, LVI, micropapillary component, and solid component, were also less frequent in the sublobar group (Table 2).

Marked differences were also observed in lymph-node assessment. Documented lymph-node assessment was available for 852 of 872 patients (97.7%), including 750 of 755 patients undergoing lobectomy (99.3%) and 102 of 117 undergoing sublobar resection (87.2%). The remaining 20 patients were clinically staged as N0 but had no structured record of nodal assessment. Median numbers of examined lymph-node stations were seven after lobectomy and three after sublobar resection (absolute SMD = 1.610), and the corresponding median examined-node counts were 11 and five (absolute SMD = 1.120). Systematic LND was performed in 98.7% and 60.7% of patients, respectively (absolute SMD = 1.071). Surgery year also showed meaningful imbalance (absolute SMD = 0.524). These large baseline differences underscore the nonrandom nature of procedure selection and the potential for residual confounding despite multivariable and propensity-score adjustment (Table 2).

**Table 2:** Baseline clinicopathological and lymph-node assessment characteristics of the clinicopathological cohort according to surgical extent.

Characteristic	Overall (N = 872)	Lobectomy (N = 755)	Sublobar (N = 117)	*p* Value	SMD
Demographic and treatment-era characteristics					
Age, years	62.0 (56.0–67.0)	61.0 (55.0–66.0)	64.0 (56.0–69.0)	0.008	0.216
Sex, *n* (%)				0.481	0.081
Female	537 (61.6)	461 (61.1)	76 (65.0)		
Male	335 (38.4)	294 (38.9)	41 (35.0)		
Year of surgery	2019 (2017–2020)	2019 (2017–2020)	2020 (2019–2020)	<0.001	0.524
Tumor and stage characteristics					
Tumor size, cm	1.6 (1.2–2.3)	1.8 (1.3–2.5)	1.1 (0.9–1.5)	<0.001	0.957
Pathologic stage, *n* (%)				0.012	0.282
IA	652 (74.8)	553 (73.2)	99 (84.6)		
IB	220 (25.2)	202 (26.8)	18 (15.4)		
Lymph-node assessment					
Lymph-node stations examined	7 (5–7)	7 (6–7)	3 (2–5)	<0.001	1.610
Lymph nodes examined	10 (8–14)	11 (8–14)	5 (2–8)	<0.001	1.120
Systematic lymph-node dissection, *n* (%)				<0.001	1.071
No	56 (6.4)	10 (1.3)	46 (39.3)		
Yes	816 (93.6)	745 (98.7)	71 (60.7)		
Any documented lymph-node assessment, *n* (%)				<0.001	0.500
No	20 (2.3)	5 (0.7)	15 (12.8)		
Yes	852 (97.7)	750 (99.3)	102 (87.2)		
Core high-risk pathological features					
Visceral pleural invasion, *n* (%)				0.004	0.336
Absent	720 (82.6)	612 (81.1)	108 (92.3)		
Present	152 (17.4)	143 (18.9)	9 (7.7)		
Lymphovascular invasion, *n* (%)				<0.001	0.494
Absent	628 (72.0)	524 (69.4)	104 (88.9)		
Present	244 (28.0)	231 (30.6)	13 (11.1)		
Micropapillary component, *n* (%)				<0.001	0.587
Absent	547 (62.7)	448 (59.3)	99 (84.6)		
Present	325 (37.3)	307 (40.7)	18 (15.4)		
Solid component, *n* (%)				0.024	0.267
Absent	750 (86.0)	641 (84.9)	109 (93.2)		
Present	122 (14.0)	114 (15.1)	8 (6.8)		
Core-HRPF burden, *n* (%)				<0.001	0.763
0	398 (45.6)	309 (40.9)	89 (76.1)		
1	204 (23.4)	191 (25.3)	13 (11.1)		
≥2	270 (31.0)	255 (33.8)	15 (12.8)		
Participating institution, *n* (%)					
Institution 1	805 (92.3)	699 (92.6)	106 (90.6)		
Institution 2	42 (4.8)	37 (4.9)	5 (4.3)		
Institution 3	13 (1.5)	8 (1.1)	5 (4.3)		
Institution 4	12 (1.4)	11 (1.5)	1 (0.9)		

Abbreviations: Core-HRPF, core high-risk pathological feature; IQR, interquartile range; LND, lymph node dissection; LVI, lymphovascular invasion (lymphatic or vascular); SMD, standardized mean difference; VPI, visceral pleural invasion (PL1 or PL2). Note: Continuous variables are presented as median (IQR) and categorical variables as n (%). *p* values were calculated using the Wilcoxon rank-sum test for continuous variables and the chi-square or Fisher exact test for categorical variables, as appropriate. Absolute SMDs quantify imbalance between lobectomy and sublobar resection; values > 0.10 indicate meaningful imbalance. The cohort was single-center dominant, with the lead institution contributing 805 of 872 patients (92.3%). SMDs are not presented for the multicategory participating-institution variable.

### 3.4. Association of Surgical Extent with DFS and OS

**Figure 3:**
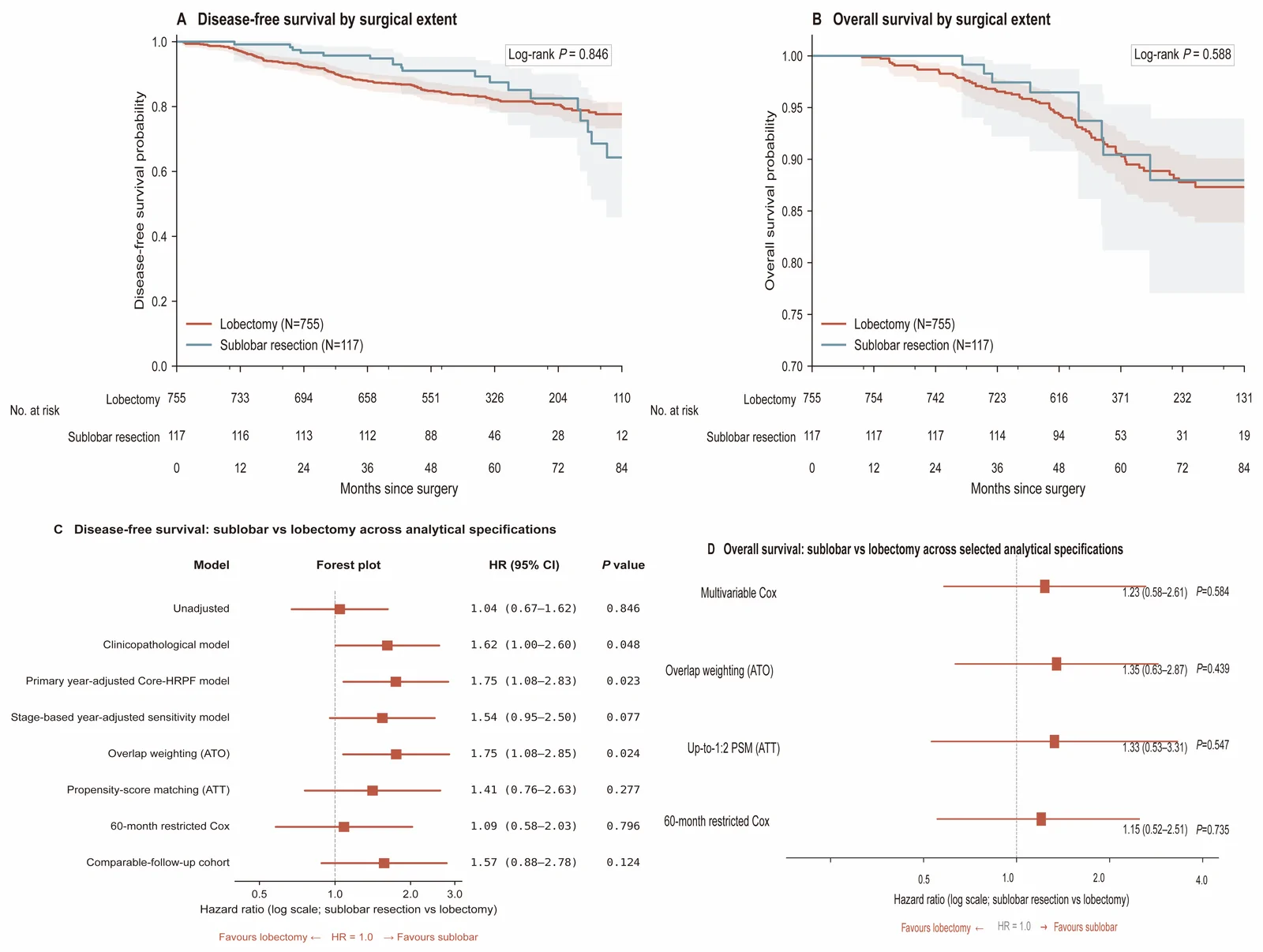
Disease-free and overall survival according to surgical extent in the clinicopathological cohort. (**A**) Unadjusted Kaplan–Meier curves for DFS after lobectomy and sublobar resection, displayed through 84 months with numbers at risk. (**B**) Unadjusted Kaplan–Meier curves for OS through 84 months with numbers at risk. (**C**) Forest plot of the association between sublobar resection and DFS across alternative analytical specifications, including the unadjusted model, clinicopathological model, primary year-adjusted Core-HRPF model, stage-based year-adjusted sensitivity model, overlap weighting, propensity-score matching, 60-month restricted Cox analysis, and comparable-follow-up cohort analysis. Lobectomy was the reference group. HRs > 1 indicate a higher DFS event hazard associated with sublobar resection; whereas, HRs < 1 indicate a lower hazard. The 84-month display horizon was selected because the sublobar risk set became sparse thereafter; full available follow-up was retained in Cox models unless a restricted analysis is explicitly indicated. (**D**) Forest plot of selected OS estimates from the primary postoperative model, overlap weighting, propensity-score matching, and the 60-month restricted Cox analysis. Abbreviations: ATT, average treatment effect among the treated; ATO, average treatment effect in the overlap population; CI, confidence interval; Core-HRPF, Core high-risk pathological feature; DFS, disease-free survival; HR, hazard ratio; OS, overall survival.

During follow-up, 166 DFS events and 81 deaths occurred. Unadjusted analyses showed no statistically clear differences between lobectomy and sublobar resection. The unadjusted DFS HR was 1.04 (95% CI 0.67–1.62; *p* = 0.846), and the corresponding OS HR was 0.83 (95% CI 0.41–1.65; *p* = 0.588) (Figure 3A,B; Table 3).

In the postoperative clinicopathological adjustment model (age, sex, tumor maximum diameter, Core-HRPF burden, and surgery year), sublobar resection was associated with a higher average follow-up DFS hazard (adjusted HR 1.75, 95% CI 1.08–2.83; *p* = 0.023). In contrast, the adjusted OS HR was 1.23 (95% CI 0.58–2.61; *p* = 0.584). The clinicopathological model without surgery year yielded a DFS HR of 1.62 (95% CI 1.00–2.60; *p* = 0.048), and the stage-based sensitivity model yielded a DFS HR of 1.54 (95% CI 0.95–2.50; *p* = 0.077) (Table 3; Figure 3C).

The DFS association varied across alternative analytical specifications. Overlap weighting yielded an HR of 1.75 (95% CI 1.08–2.85; *p* = 0.024); whereas, propensity-score matching yielded an HR of 1.41 (95% CI 0.76–2.63; *p* = 0.277). When follow-up was restricted to 60 months, the DFS HR was 1.09 (95% CI 0.58–2.03; *p* = 0.796), while restriction to the comparable-follow-up cohort yielded an HR of 1.57 (95% CI 0.88–2.78; *p* = 0.124). Thus, the direction of several estimates remained above unity, but statistical support and effect magnitude varied across analytical approaches (Table 3; Figure 3).

### 3.5. Non-Proportional DFS Association and Follow-Up Sensitivity Analyses

In the primary DFS model, the proportional-hazards assumption was violated for surgical extent (Schoenfeld residual *p* = 0.012). During 0–36 months postoperatively, the DFS HR for sublobar resection was 0.67 (95% CI 0.26–1.71; *p* = 0.402); during 36–60 months it was 1.84 (95% CI 0.84–4.01; *p* = 0.125); the >60-month estimate increased substantially but was based on a sparse late risk set (372 patients at risk at 60 months and 29 subsequent DFS events) and was therefore considered exploratory. Detailed interval-specific estimates are provided in the Supplementary Materials.

When follow-up was administratively restricted to 60 months, the sublobar-versus-lobectomy HR was 1.09 (95% CI 0.58–2.03; *p* = 0.796) for DFS and 1.15 (95% CI 0.52–2.51; *p* = 0.735) for OS. The covariate-adjusted 60-month RMST difference (sublobar resection minus lobectomy) was 1.37 months (95% CI −0.73 to 3.46; *p* = 0.200), providing no statistically clear evidence of a difference in average 60-month DFS (Table S3).

### 3.6. Recurrence Patterns and Locoregional Recurrence

Among 872 patients, 154 recurrences, 12 pre-recurrence deaths, and 706 censored alive-without-recurrence were recorded. By surgical extent: lobectomy group (N = 755) had 133 recurrences; sublobar group (N = 117) had 21. Locoregional recurrence was recorded in 97 patients (lobectomy 79, sublobar 18) and distant metastasis in 59 (lobectomy 58, sublobar 1); 19 patients had both locoregional and distant recurrence recorded, and 17 had recurrence without locoregional or distant classification. As site-specific recurrence dates were not available, the temporal sequence of locoregional and distant events could not be determined for patients with both. In the site-specific competing-risk models, recurrences without involvement of the target site and pre-recurrence deaths were treated as competing events, yielding 69 competing events for the locoregional analysis and 107 for the distant analysis.

**Figure 4:**
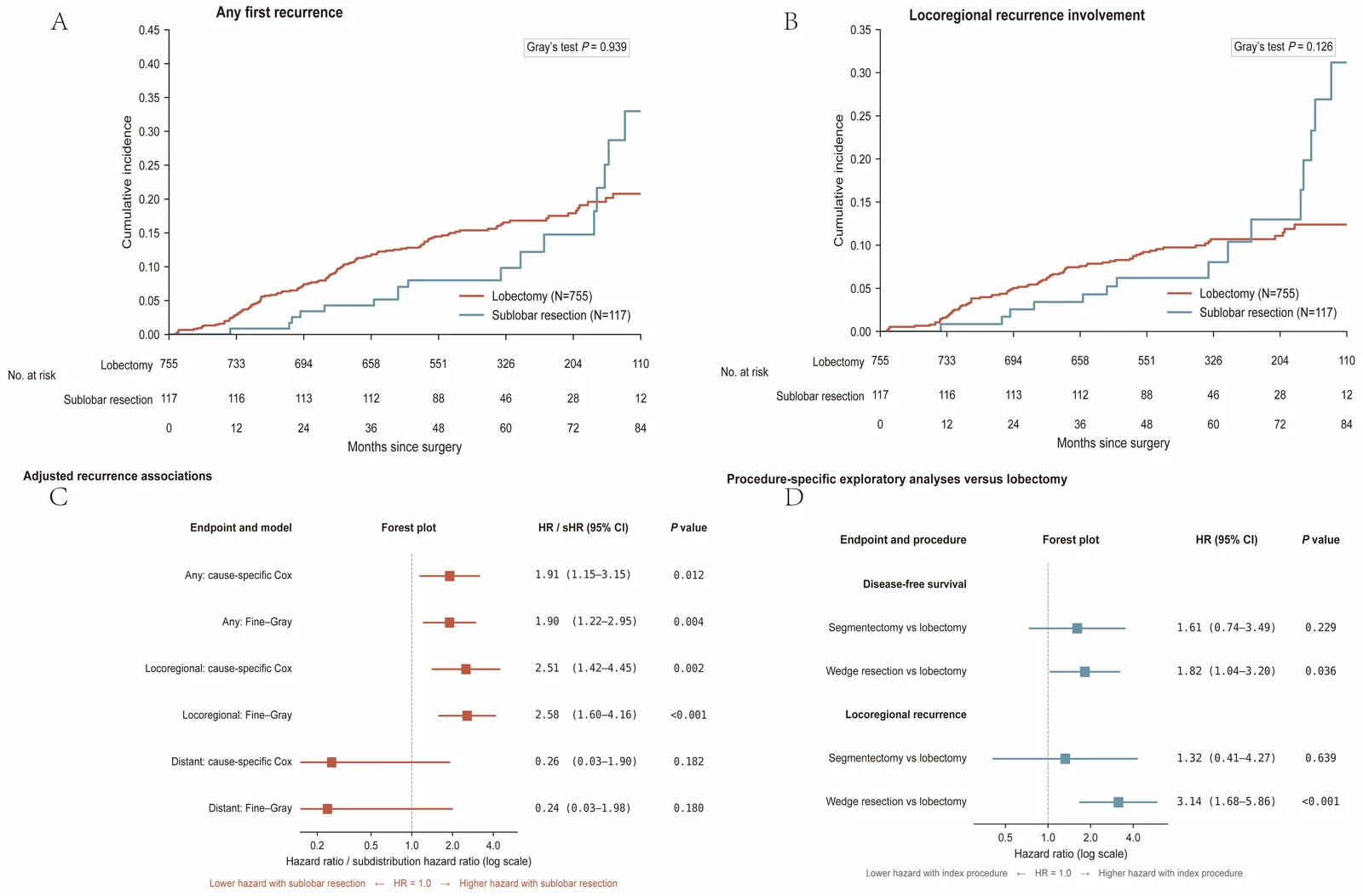
Recurrence patterns and procedure-specific exploratory analyses in the clinicopathological cohort. (**A**) Cumulative incidence of any first recurrence by surgical extent. (**B**) Cumulative incidence of locoregional first recurrence. (**C**) Adjusted cause-specific Cox and Fine–Gray estimates for any recurrence and for locoregional and distant recurrence involvement. (**D**) Exploratory procedure-specific DFS and locoregional recurrence estimates for segmentectomy and wedge resection, each compared with lobectomy. The distant-recurrence estimates in panel (**C**) were based on only one distant recurrence in the sublobar group and should not be interpreted as evidence of a protective association. Abbreviations: CI, confidence interval; Core-HRPF, Core high-risk pathological feature; DFS, disease-free survival; HR, hazard ratio; sHR, subdistribution hazard ratio.

After adjusting for age, sex, tumor maximum diameter, Core-HRPF burden, and surgery year, sublobar resection was associated with higher any-recurrence risk: cause-specific Cox HR 1.91 (95% CI 1.15–3.15; *p* = 0.012) and Fine–Gray sHR 1.90 (95% CI 1.22–2.95; *p* = 0.004) (Table 4; Figure 4). In exploratory procedure-specific analyses, the locoregional recurrence estimate was imprecise after segmentectomy (HR 1.32, 95% CI 0.41–4.27; *p* = 0.639) but was higher after wedge resection (HR 3.14, 95% CI 1.68–5.86; *p* < 0.001) (Table 4; Figure 4D). These estimates were exploratory because of the limited procedure-specific sample sizes and event counts.

By recurrence pattern, the adjusted recurrence association was predominantly locoregional. The locoregional recurrence cause-specific HR was 2.51 (95% CI 1.42–4.45; *p* = 0.002) and the Fine–Gray sHR was 2.56 (95% CI 1.59–4.14; *p* < 0.001). For distant recurrence, the cause-specific HR was 0.26 (95% CI 0.03–1.90; *p* = 0.182) and the Fine–Gray sHR was 0.24 (95% CI 0.03–1.98; *p* = 0.180). Because only one distant recurrence occurred in the sublobar group, these distant-recurrence estimates were highly imprecise and should not be interpreted as evidence of a protective association (Table 4; Figure 4).

### 3.7. Lymph-Node Assessment Sensitivity Analysis

Given the substantially less extensive lymph-node assessment in the sublobar group, the association between surgical extent and outcomes was re-evaluated in cohorts restricted according to prespecified nodal-assessment criteria.

Among 816 patients who underwent systematic LND (lobectomy 745, sublobar 71; 157 DFS events), the adjusted DFS HR was 2.11 (95% CI 1.25–3.58; *p* = 0.005). Among 822 patients with ≥3 nodal stations examined (748 lobectomy, 74 sublobar; 159 events), the DFS HR was 1.97 (95% CI 1.16–3.34; *p* = 0.012). Among 770 patients with ≥6 lymph nodes examined (721 lobectomy, 49 sublobar; 150 events), the DFS HR was 2.01 (95% CI 1.09–3.71; *p* = 0.026). Among 766 patients meeting both criteria (720 lobectomy, 46 sublobar; 148 events), the DFS HR was 1.80 (95% CI 0.93–3.48; *p* = 0.083). The restricted-cohort DFS HRs therefore ranged from 1.80 to 2.11. Corresponding OS analyses showed no statistically clear differences (Table 4).

When lymph-node indicators were directly added to the primary DFS model, effect estimates were attenuated to varying degrees: adding nodal-station count yielded HR 1.64 (*p* = 0.088); adding examined-node count yielded HR 1.69 (*p* = 0.040); adding systematic LND yielded HR 1.67 (*p* = 0.067); and adding both station and node counts yielded HR 1.64 (*p* = 0.088) (Table 4). These sensitivity analyses evaluate robustness to measured differences in nodal assessment but cannot disentangle surgical extent from nodal-staging quality or potential stage migration.

### 3.8. Core-HRPF Burden and Long-Term Prognosis

Among 872 primary analysis patients, 398 had no Core-HRPF, 204 had 1 Core-HRPF, and 270 had ≥2 Core-HRPF.

**Figure 5:**
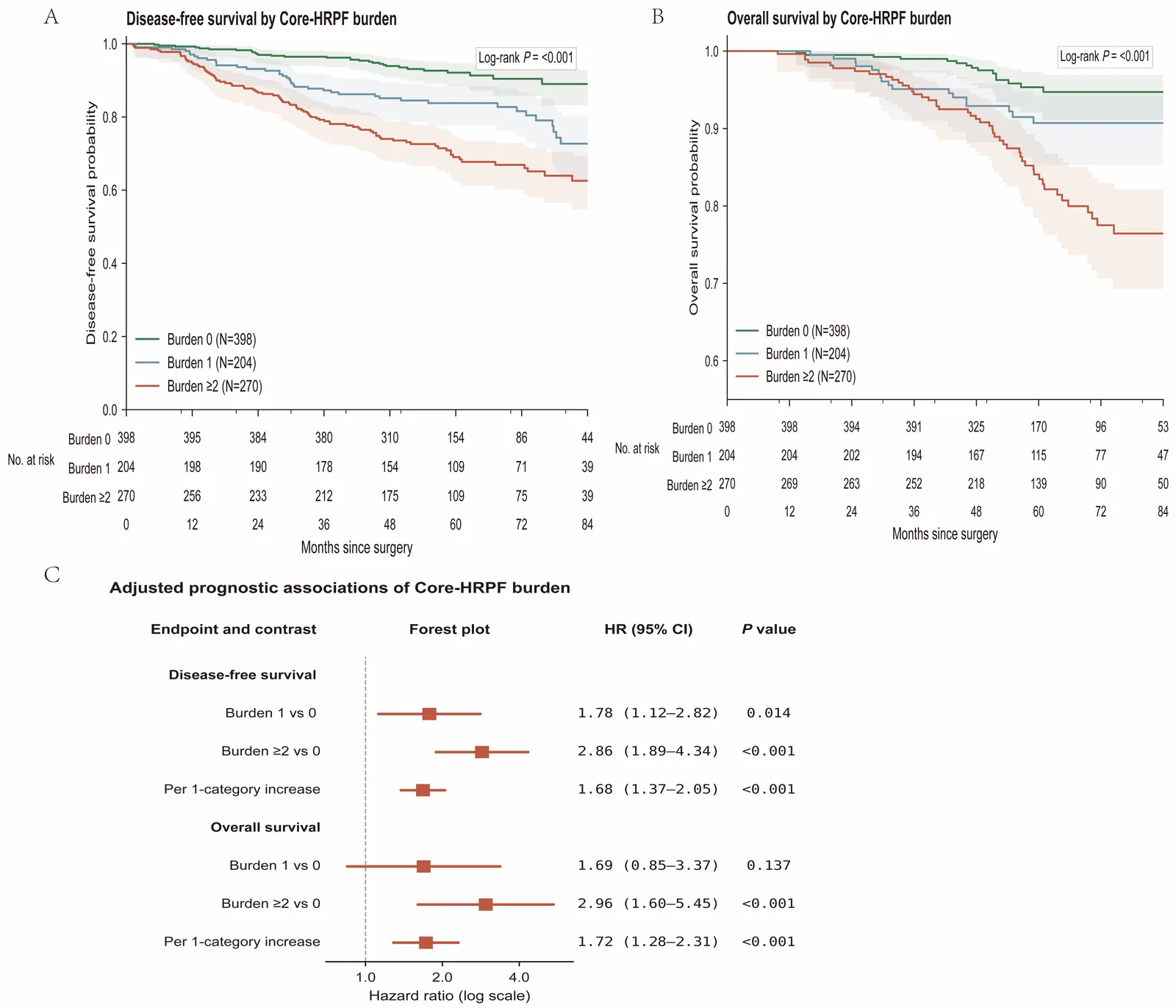
Core-HRPF burden and postoperative prognosis. (**A**) Kaplan–Meier DFS curves according to Core-HRPF burden 0, 1, and ≥2. (**B**) Kaplan–Meier OS curves according to Core-HRPF burden. (**C**) Adjusted HRs for Core-HRPF burden 1 versus 0 and ≥2 versus 0 for DFS and OS. Models were adjusted for age, sex, maximum tumor diameter, and surgery year. Ordinal analyses demonstrated increasing hazards per one-category increase in Core-HRPF burden for both DFS (HR 1.68, 95% CI 1.37–2.05; *p* < 0.001) and OS (HR 1.72, 95% CI 1.28–2.31; *p* < 0.001), as reported in Table 5. Abbreviations: CI, confidence interval; Core-HRPF, Core high-risk pathological feature; DFS, disease-free survival; HR, hazard ratio; OS, overall survival.

Core-HRPF burden showed a graded relationship with long-term outcome. Five-year DFS was 92.1% (95% CI 88.6–94.6) for burden 0, 83.8% (95% CI 77.8–88.3) for burden 1, and 69.0% (95% CI 62.8–74.5) for burden ≥2. Corresponding five-year OS was 95.3% (95% CI 92.1–97.3), 90.7% (95% CI 85.4–94.2), and 84.1% (95% CI 78.6–88.3), respectively (Table 5; Figure 5).

After adjustment for age, sex, tumor size, and surgery year, with burden 0 as the reference, burden 1 was associated with DFS HR 1.78 (95% CI 1.12–2.82; *p* = 0.014) and burden ≥2 with DFS HR 2.86 (95% CI 1.89–4.34; *p* < 0.001). Corresponding OS HRs were 1.69 (95% CI 0.85–3.37; *p* = 0.137) and 2.96 (95% CI 1.60–5.45; *p* < 0.001). Treating Core-HRPF burden as an ordinal variable showed a significant trend for both DFS (HR per one-category increase 1.68, 95% CI 1.37–2.05; *p* for trend <0.001) and OS (HR 1.72, 95% CI 1.28–2.31; *p* for trend <0.001). The surgery-by-Core-HRPF interaction was not statistically significant for DFS (*p* = 0.518) (Table 5; Figure 5). A sensitivity model replacing the burden count with the four individual Core-HRPF components yielded a similar adjusted DFS estimate for sublobar resection (HR 1.66, 95% CI 1.02–2.69; *p* = 0.039). Overall, Core-HRPF burden showed graded postoperative prognostic associations; whereas, no statistically clear interaction with surgical extent was detected.

### 3.9. Exploratory Procedure-Specific Survival Analyses

In exploratory procedure-specific analyses, the adjusted DFS estimate was 1.61 (95% CI 0.74–3.49) for segmentectomy and 1.82 (95% CI 1.04–3.20) for wedge resection, each compared with lobectomy. The corresponding 60-month restricted DFS estimates were 1.60 (95% CI 0.69–3.68) and 0.81 (95% CI 0.35–1.90), respectively. Adjusted OS estimates were also imprecise, particularly for segmentectomy (Table 6).

## 4. Discussion

### 4.1. Principal Findings

This study used a two-stage complementary real-world framework to distinguish population-level outcome associations from the clinicopathological and surgical-quality context in which those signals arise. The central finding is not that sublobar resection is uniformly inferior to lobectomy, but that the real-world label “sublobar resection” combines procedures and implementation conditions with materially different outcome patterns.

In SEER, the binary sublobar-versus-lobectomy comparison showed statistically significant differences in five-year OS and lung cancer-specific death, but procedure-specific modeling demonstrated marked heterogeneity: segmentectomy showed no statistically clear OS difference from lobectomy; whereas, wedge resection showed the largest and most consistent adverse associations. Further adjustment for examined lymph-node count attenuated the binary estimates. These findings support interpreting the SEER association as reflecting a mixture of procedure type, measured and unmeasured case mix, and nodal-assessment quality rather than as a uniform effect of all sublobar resections.

Th clinicopathological cohort provided a complementary explanation for this heterogeneity. Patients selected for sublobar resection had smaller tumors, earlier pathological stage, and lower Core-HRPF burden, but also substantially less extensive nodal evaluation. Unadjusted DFS and OS were not statistically different. After postoperative clinicopathological adjustment, the average follow-up DFS estimate shifted above 1, while OS remained statistically unclear. Importantly, the DFS association was non-proportional over time, the 60-month restricted Cox analysis showed no clear difference, and the covariate-adjusted 60-month RMST analysis likewise showed no statistically clear difference. Recurrence analyses indicated that the adjusted recurrence association was predominantly locoregional.

### 4.2. Differences from Randomized Trials and Consistency of the Locoregional Recurrence Signal

The JCOG0802/WJOG4607L and CALGB/Alliance 140503 trials significantly altered the evidence base for surgical extent in small peripheral NSCLC [2,3]. JCOG0802/WJOG4607L compared anatomical segmentectomy with lobectomy in patients with peripheral NSCLC ≤ 2 cm, showing non-inferior and superior overall survival for segmentectomy; five-year relapse-free survival was nearly identical between groups, but the segmentectomy group had a higher local recurrence proportion (10.5% vs. 5.4%) [2,26]. CALGB/Alliance 140503 demonstrated that, in patients with peripheral tumors ≤ 2 cm and intraoperatively confirmed node-negative disease, sublobar resection was non-inferior to lobectomy in DFS, with comparable OS [3,4].

In contrast, the primary postoperative model in the clinicopathological cohort yielded a higher average follow-up DFS hazard, while recurrence analyses showed a higher recurrence risk after sublobar resection. However, this difference does not imply that the present study refutes the RCT evidence. The RCTs addressed whether a prespecified sublobar resection strategy could achieve target oncological efficacy under rigorous selection, standardized nodal staging, and controlled surgical conditions. This study evaluates what happens when sublobar resection enters routine clinical practice, covering more complex patient selection, procedure composition, and surgical quality conditions.

More notably, despite the model-dependent DFS findings, the locoregional recurrence association observed in this study was directionally consistent with the local-control concern identified in the randomized trials. In CALGB/Alliance 140503, locoregional recurrence rates were 13.4% for sublobar resection versus 10.0% for lobectomy [3]. In the present study, cause-specific Cox and Fine–Gray analyses similarly localized the adjusted recurrence signal predominantly to locoregional recurrence rather than distant metastasis. This pattern can be interpreted in the context of previous studies linking local control after sublobar resection to surgical margins, procedure type, and invasive pathological features [27,28,29,30,31].

### 4.3. “Sublobar Resection” Is Not a Homogeneous Surgical Exposure

One of the most informative findings of the SEER analysis was the separation of anatomical segmentectomy from wedge resection. Segmentectomy showed no statistically clear difference from lobectomy in OS; whereas, wedge resection showed more pronounced adverse associations with both all-cause and lung cancer-specific mortality. The cause-specific and Fine–Gray estimates for lung cancer death after segmentectomy were modestly above 1 and had *p* values close to 0.05; because multiple endpoints and models were examined without multiplicity correction, these estimates should be interpreted cautiously rather than as definitive evidence of excess disease-specific mortality. Overall, combining segmentectomy and wedge resection into a single “sublobar resection” exposure can obscure clinically important procedural heterogeneity [5,32,33]. Exploratory procedure-specific analyses in the clinicopathological cohort were also compatible with procedural heterogeneity, although estimates for segmentectomy and wedge resection were imprecise and varied according to endpoint and follow-up horizon.

Anatomical segmentectomy requires management of the corresponding segmental bronchus, vasculature, and intersegmental plane, typically allowing for anatomically appropriate margins and concurrent hilar and mediastinal lymph node assessment when tumor location is suitable. Wedge resection is a non-anatomical parenchymal excision whose margin length, depth, and lymph node evaluation depend more on specific lesion location, patient status, and surgeon practice [34,35]. However, the unfavorable wedge-resection association in SEER cannot be directly attributed to the procedure itself, because real-world wedge resection may be preferentially selected for patients with advanced age, impaired pulmonary reserve, frailty, comorbidities, or limited tolerance for anatomical resection—factors that cannot be adequately controlled in SEER [36,37].

### 4.4. Patient Selection and Postoperative Clinicopathological Adjustment

In the clinicopathological cohort, patients undergoing sublobar resection had smaller tumors, earlier pathological stage, and lower Core-HRPF burden. This favorable pathological case mix helps explain why the unadjusted survival curves did not show an adverse sublobar signal. After adjustment for age, sex, tumor size, Core-HRPF burden, and surgery year, the DFS HR increased from approximately 1.0 to 1.75, illustrating the extent to which postoperative pathological case mix influenced the adjusted estimate.

Core-HRPF, however, is a postoperative variable and cannot represent information available at the time of surgical decision-making. The Core-HRPF-containing model therefore standardizes prognosis at a common final pathological risk level but does not emulate preoperative treatment assignment and should not be interpreted causally. Risk-balanced analyses were not fully concordant: overlap weighting yielded an estimate similar to the primary postoperative model; whereas, the propensity-score-matched estimate was imprecise and compatible with no clear difference.

### 4.5. Recurrence Patterns and Lymph Node Evaluation

DFS is a composite outcome of recurrence and death. Observing only an elevated DFS HR cannot distinguish whether outcome differences arise from tumor recurrence, non-tumor death, or follow-up and outcome recording differences. In this study, only 12 pre-recurrence deaths occurred versus 154 recurrences; after treating pre-recurrence deaths as competing events, sublobar resection remained associated with higher any recurrence risk. This finding suggests that the multivariable DFS association was unlikely to be explained primarily by non-recurrence death.

More importantly, the adjusted recurrence association was predominantly locoregional. The cause-specific HR and Fine–Gray sHR for locoregional recurrence were both above unity and statistically significant, while distant recurrence showed no statistically significant increase. However, with only one distant recurrence event in the sublobar group, the confidence interval is extremely wide, and “not significantly increased” does not equate to “no increase”; the possibility that tumor biology simultaneously influences both local and distant recurrence cannot be excluded.

This result is consistent with the higher local recurrence after segmentectomy reported in JCOG0802/WJOG4607L [2]. Possible explanations include inadequate margin distance, insufficient margin-to-tumor diameter ratio, higher proportion of non-anatomical wedge resection, inadequate intrapulmonary lymphatic drainage management, or occult nodal metastasis, but these factors were not completely and systematically recorded in this database [26,27,34,35,38]. Lymph-node assessment differences also represent important context. In sensitivity analyses restricted according to nodal-assessment criteria, the DFS estimates remained above unity but were variably precise; with additional adjustment for nodal measures, the estimates were modestly attenuated and also varied in precision. These analyses do not allow the effects of surgical extent to be disentangled from nodal-staging quality or potential stage migration [17,18].

### 4.6. Prognostic Significance of Core-HRPF

Even among patients with lung adenocarcinoma measuring ≤3 cm and node-negative disease, long-term prognosis was heterogeneous. Five-year DFS and OS declined progressively across Core-HRPF burden categories 0, 1, and ≥2. The adjusted ordinal trend was statistically significant for both DFS (HR per category 1.68; *p* for trend <0.001) and OS (HR 1.72; *p* for trend <0.001), supporting a graded prognostic relationship rather than an isolated contrast between categories.

This result indicates that tumor diameter and node-negative status are insufficient to fully define biologically low-risk patients. VPI and LVI reflect pleural and lymphovascular invasive potential; whereas, micropapillary and solid growth represent high-grade architectural patterns that have consistently been associated with recurrence and unfavorable survival in resected lung adenocarcinoma [6,7,8,9,20,39,40,41,42,43,44,45,46]. Notably, the prognostic relevance of micropapillary and solid components is not confined to predominant tumors; even minor high-grade components have been associated with lymph-node metastasis and inferior recurrence-free outcomes [42,43,44]. Integrating these features into a risk burden may help identify patients with higher recurrence risk under traditional anatomical staging. However, as the primary model did not simultaneously include pathological stage and Core-HRPF, this study could not formally test the incremental value of Core-HRPF beyond pathological staging. Moreover, replacing the burden count with the four individual Core-HRPF components yielded a similar surgical-extent estimate, suggesting that the primary DFS estimate was not solely dependent on the equal-weight burden formulation.

Core-HRPF therefore functions in this study as a postoperative prognostic descriptor rather than a validated predictive treatment-selection biomarker. Although previous studies have suggested that the prognostic impact of invasive histological features may vary according to the extent of pulmonary resection [47,48,49], our surgery-by-Core-HRPF interaction was not statistically significant for DFS (*p* = 0.518), and the present cohort was not powered to establish treatment-effect heterogeneity. Accordingly, the absence of a statistically significant interaction should not be interpreted as evidence that surgical effects are identical across Core-HRPF strata.

Yang et al. reported that the oncologic adequacy of resection extent varied according to tumor biology in node-negative invasive lung adenocarcinoma measuring ≤2 cm: low-risk tumors had low local-recurrence rates across resection types; whereas, high-risk tumors showed progressively less favorable outcomes with lesser resection extent [10]. These findings provide external context for the importance of postoperative pathological heterogeneity. Our Core-HRPF analysis, however, addressed prognosis rather than procedure-specific treatment benefit, because no statistically significant surgery-by-Core-HRPF interaction was detected for DFS (*p* = 0.518). Core-HRPF should therefore be interpreted as a postoperative prognostic descriptor that may provide additional postoperative risk-stratification context and inform surveillance planning, rather than as evidence that one surgical extent is superior within a particular Core-HRPF stratum.

### 4.7. Strengths, Limitations, and Clinical Implications

This study has several strengths. First, the two-stage framework assigns distinct roles to the two data sources: SEER provides a large population-level outcome benchmark; whereas, the clinicopathological cohort provides detailed pathological, nodal, and recurrence information for interpretation. Second, recurrence was separated into locoregional and distant patterns with pre-recurrence death treated as a competing event. Third, the analysis transparently reports unadjusted and multivariable estimates, lymph-node assessment restrictions, and 60-month Cox/RMST analyses rather than relying on a single favorable model.

Important limitations exist. First, both SEER and the clinicopathological cohort are observational data with residual unmeasured confounding. The databases lack or incompletely record pulmonary function, comorbidities, performance status, frailty, surgical selection rationale, and patient preferences. Second, 460 of 1332 screened patients were excluded, including 237 (17.8%) for missing or invalid survival data, potentially introducing selection bias. Third, complete preoperative imaging information is lacking. Fourth, the in-house database lacks standardized margin distance, margin-to-tumor diameter ratio, and wedge resection depth. Fifth, although a center variable was available, one center contributed 805 of 872 patients (92.3%), making the cohort effectively single-center-dominant and precluding meaningful center-stratified analyses; this limits generalizability to other institutions. In addition, 20 patients lacked structured nodal-assessment documentation and were classified as N0 on clinical staging, leaving some residual uncertainty regarding nodal staging. Sixth, the proportional-hazards assumption was violated for DFS, and late follow-up estimates were based on small risk sets and were unstable; accordingly, long-term time-specific estimates should be interpreted cautiously. Seventh, among 117 sublobar resection patients, 112 had tumors ≤2 cm, so the sublobar comparison primarily reflects ≤2 cm tumor real-world practice. Eighth, pathological features were assessed at individual participating institutions without central pathology review, and recurrence patterns were derived from routine clinical records without centralized radiological review; inter-institutional variation in pathological and recurrence assessment therefore cannot be excluded. Ninth, multiple supportive and exploratory analyses were performed without multiplicity correction. Tenth, the surgery-by-Core-HRPF interaction analysis was likely underpowered given the limited number of sublobar resection patients and events, and the non-significant result should not be interpreted as evidence of no effect modification.

The conceptual contribution of this study is to reframe the translation of randomized evidence from a binary question—“sublobar resection versus lobectomy”—to an implementation question: which sublobar procedure is performed, in which patient, with what nodal assessment and oncological quality, and against what postoperative pathological risk. Clinically, the data argue against treating segmentectomy and wedge resection as interchangeable. When sublobar resection is selected, oncological quality should be documented through procedure type, adequate hilar and mediastinal nodal assessment, parenchymal margin metrics—including absolute margin distance and the margin-to-tumor ratio when available—and recurrence pattern [17,18,19,34,35,49,50]. Core-HRPF can add postoperative prognostic context and may help inform postoperative risk assessment and surveillance planning, but it should not be used as a preoperative treatment-selection rule on the basis of this study alone.

## 5. Conclusions

By combining a population registry with a pathology-rich multi-institutional, single-center-dominant clinicopathological cohort, this study identifies heterogeneity in the real-world implementation of sublobar resection rather than a uniform disadvantage of sublobar resection. In SEER, unfavorable mortality associations were more pronounced for wedge resection than for segmentectomy and were attenuated after adjustment for examined lymph-node count; whereas, segmentectomy showed no statistically clear OS difference from lobectomy. In the clinicopathological cohort, the DFS association varied across analytical specifications: the primary postoperative and overlap-weighted models yielded higher DFS hazards; whereas, propensity-score matching, fixed-horizon analysis, and the comparable-follow-up analysis remained statistically inconclusive. The adjusted recurrence association was predominantly locoregional and was more evident after wedge resection in exploratory procedure-specific analyses. Core-HRPF provided graded postoperative prognostic information; whereas, no statistically clear interaction with surgical extent was detected. These findings support interpreting sublobar resection as a procedure- and quality-dependent real-world exposure, with attention to procedure type, nodal assessment, and postoperative pathological risk, rather than treating all sublobar resections as a single category or inferring a causal disadvantage of sublobar resection per se.

## Figures and Tables

**Table 1:** Associations between surgical procedure and overall survival and lung cancer-specific death in the SEER strict node-assessed N0M0 (“pN0-like”) cohort.

Section	Analysis	Comparison	Population	OS HR (95% CI)	OS *p*	Cause-Specific Death HR (95% CI)	Cause-Specific *p*	Fine–Gray sHR (95% CI)	FG *p*
Binary surgical-extent comparison	Multivariable models	Sublobar resection vs. lobectomy	N = 22,864	1.31 (1.23–1.38)	<0.001	1.38 (1.27–1.51)	<0.001	1.33 (1.22–1.45)	<0.001
Binary surgical-extent comparison	Overlap weighting (ATO)	Sublobar resection vs. lobectomy	N = 22,864; ESS = 13,821.3	1.29 (1.22–1.37)	<0.001	1.36 (1.25–1.48)	<0.001	—	—
Binary surgical-extent comparison	Up-to-1:2 PSM (ATT)	Sublobar resection vs. lobectomy	N = 13,560; 4567 matched sets	1.31 (1.23–1.39)	<0.001	1.38 (1.25–1.51)	<0.001	—	—
Procedure-specific comparison	Multivariable models	Segmentectomy vs. lobectomy	N = 22,864	1.07 (0.97–1.19)	0.174	1.17 (1.01–1.35)	0.040	1.16 (1.00–1.34)	0.047
Procedure-specific comparison	Multivariable models	Wedge resection vs. lobectomy	N = 22,864	1.42 (1.33–1.51)	<0.001	1.49 (1.35–1.64)	<0.001	1.41 (1.28–1.55)	<0.001
Lymph-node-count sensitivity	Same-cohort reference (no LN adjustment)	Sublobar resection vs. lobectomy	N = 21,649	1.29 (1.21–1.37)	<0.001	1.39 (1.27–1.51)	<0.001	—	—
Lymph-node-count sensitivity	Additional adjustment for examined lymph-node count	Sublobar resection vs. lobectomy	N = 21,649	1.19 (1.11–1.26)	<0.001	1.26 (1.15–1.38)	<0.001	—	—
Lymph-node-count sensitivity	Additional adjustment for examined lymph-node count	Segmentectomy vs. lobectomy	N = 21,649	1.02 (0.91–1.13)	0.762	1.12 (0.96–1.30)	0.160	—	—
Lymph-node-count sensitivity	Additional adjustment for examined lymph-node count	Wedge resection vs. lobectomy	N = 21,649	1.27 (1.18–1.36)	<0.001	1.33 (1.20–1.48)	<0.001	—	—

Abbreviations: CI, confidence interval; FG, Fine–Gray; HR, hazard ratio; OS, overall survival; sHR, subdistribution hazard ratio. Note: The binary analysis combines segmentectomy and wedge resection into sublobar resection. The procedure-specific analysis separately compares segmentectomy and wedge resection versus lobectomy. The lymph-node-count sensitivity analyses were restricted to patients with an exact examined-node count (N = 21,649). A same-cohort reference model used the original adjustment covariates without examined-node count, and examined-node count was then added to the model to evaluate attenuation within the identical analysis population.

**Table 3:** Associations of sublobar resection with disease-free and overall survival across alternative analytical specifications in the clinicopathological cohort.

Endpoint	Model	N	Events	HR (95% CI)	*p* Value
DFS	Unadjusted	872	166	1.04 (0.67–1.62)	0.846
DFS	Clinicopathological model	872	166	1.62 (1.00–2.60)	0.048
DFS	Primary year-adjusted Core-HRPF model	872	166	1.75 (1.08–2.83)	0.023
DFS	Stage-based year-adjusted sensitivity model	872	166	1.54 (0.95–2.50)	0.077
DFS	Overlap weighting (ATO)	872	—	1.75 (1.08–2.85)	0.024
DFS	PSM (ATT)	326	49	1.41 (0.76–2.63)	0.277
DFS	60-month restricted Cox	872	137	1.09 (0.58–2.03)	0.796
DFS	Comparable-follow-up cohort	646	96	1.57 (0.88–2.78)	0.124
OS	Unadjusted	872	81	0.83 (0.41–1.65)	0.588
OS	Clinicopathological model	872	81	1.11 (0.53–2.34)	0.783
OS	Primary year-adjusted Core-HRPF model	872	81	1.23 (0.58–2.61)	0.584
OS	Stage-based year-adjusted sensitivity model	872	81	1.09 (0.51–2.31)	0.828
OS	Overlap weighting (ATO)	872	—	1.35 (0.63–2.87)	0.439
OS	PSM (ATT)	326	22	1.33 (0.53–3.31)	0.547
OS	60-month restricted Cox	872	68	1.15 (0.52–2.51)	0.735
OS	Comparable-follow-up cohort	646	48	1.08 (0.45–2.56)	0.867

Abbreviations: ATO, average treatment effect in the overlap population; ATT, average treatment effect among the treated; CI, confidence interval; Core-HRPF, core high-risk pathological feature; DFS, disease-free survival; HR, hazard ratio; OS, overall survival. Note: Lobectomy was the reference group in all analyses. The clinicopathological model adjusted for age, sex, maximum tumor diameter, and Core-HRPF burden. The primary postoperative model additionally adjusted for surgery year. The stage-based sensitivity model replaced Core-HRPF burden with pathological stage while retaining surgery year. Core-HRPF-containing models represent postoperative prognostic standardization and should not be interpreted as models of preoperative treatment allocation or causal treatment effects. The 60-month model administratively restricted follow-up to 60 months. The comparable-follow-up analysis was restricted to the prespecified common-era cohort. Propensity-score and other sensitivity analyses address measured covariate imbalance only and do not eliminate residual confounding.

**Table 4:** Recurrence patterns and lymph-node assessment sensitivity analyses in the clinicopathological cohort.

(A) Recurrence Competing Risk Analysis (N = 872; Lobectomy N = 755, Sublobar Resection N = 117)							
Recurrence Endpoint	Total Events	Lob/Sub Events	Competing	CS HR (95% CI)	CS *p*	Fine–Gray sHR (95% CI)	FG *p*
Any recurrence	154	Lob: 133/Sub: 21	12	HR 1.91 (1.15–3.15)	0.012	sHR 1.90 (1.22–2.95)	0.004
Locoregional recurrence involvement	97	Lob: 79/Sub: 18	69	HR 2.51 (1.42–4.45)	0.002	sHR 2.58 (1.60–4.16)	<0.001
Distant recurrence involvement	59	Lob: 58/Sub: 1	107	HR 0.26 (0.03–1.90)	0.182	sHR 0.24 (0.03–1.98)	0.180
**Exploratory Procedure-Specific Locoregional Recurrence Analysis**							
Comparison		Index-procedure N	LR events	CS HR (95% CI)		*p* value	
Segmentectomy vs. lobectomy		37	3	1.32 (0.41–4.27)		0.639	
Wedge resection vs. lobectomy		80	15	3.14 (1.68–5.86)		<0.001	
**(B) Lymph Node Assessment Restricted Analyses (DFS and OS)**							
**Restriction Criterion**			**N**	**Events**		**HR (95% CI)**	***p*** **Value**
At least 3 lymph-node stations examined			822	159		HR 1.97 (1.16–3.34)	0.012
At least 3 stations and at least 6 nodes			766	148		HR 1.80 (0.93–3.48)	0.083
At least 6 lymph nodes examined			770	150		HR 2.01 (1.09–3.71)	0.026
Systematic lymph-node dissection only			816	157		HR 2.11 (1.25–3.58)	0.005
≥3 stations			822	78		HR 1.22 (0.53–2.82)	0.634
≥3 stations			766	75		HR 1.84 (0.77–4.39)	0.167
≥6 nodes			770	75		HR 1.77 (0.74–4.23)	0.201
systematic LND			816	76		HR 1.30 (0.56–2.99)	0.538
**(C) Lymph Node Assessment Adjustment Analyses (DFS and OS)**							
**Adjustment Model**				**HR (95% CI)**		***p*** **Value**	
Add lymph-node count				HR 1.69 (1.02–2.79)		0.040	
Add lymph-node station count				HR 1.64 (0.93–2.89)		0.088	
Add node-count restricted cubic spline				HR 1.69 (1.02–2.79)		0.040	
Add station and node counts				HR 1.64 (0.93–2.88)		0.088	
Add systematic lymph-node dissection				HR 1.67 (0.96–2.90)		0.067	
node count				HR 1.08 (0.50–2.37)		0.838	
station count				HR 1.11 (0.48–2.60)		0.803	
node spline				HR 1.08 (0.50–2.37)		0.838	
station + node				HR 1.11 (0.47–2.58)		0.817	
systematic LND				HR 1.05 (0.46–2.40)		0.910	

Abbreviations: CI, confidence interval; CS, cause-specific; DFS, disease-free survival; FG, Fine–Gray; HR, hazard ratio; LND, lymph node dissection; sHR, subdistribution hazard ratio. Note: Recurrence models were adjusted for age, sex, maximum tumor diameter, Core-HRPF burden, and surgery year. Lobectomy was the reference group. Procedure-specific recurrence analyses were exploratory because of limited sample sizes and event counts. Lymph-node sensitivity analyses evaluate robustness to measured differences in nodal assessment and should not be interpreted as separating the independent effects of surgical extent from nodal-staging quality or potential stage migration. The distant-recurrence analysis included only one event in the sublobar group and was therefore highly imprecise. Locoregional and distant recurrence involvement were not mutually exclusive because 19 patients had both patterns recorded; site-specific dates were unavailable, and 17 recurrences had an unclassified anatomical site.

**Table 5:** Prognostic associations of the prespecified four-item Core-HRPF burden with disease-free and overall survival.

(A) Core-HRPF Burden and DFS/OS: Multivariable-Adjusted Hazard Ratios					
Endpoint	Comparison		HR (95% CI)	*p* Value	Global *p*
DFS	Core-HRPF burden 1 vs. 0		1.78 (1.12–2.82)	0.014	<0.001
DFS	Core-HRPF burden ≥ 2 vs. 0		2.86 (1.89–4.34)	<0.001	
DFS	Per one-category increase		1.68 (1.37–2.05)	<0.001	
OS	Core-HRPF burden 1 vs. 0		1.69 (0.85–3.37)	0.137	<0.001
OS	Core-HRPF burden ≥ 2 vs. 0		2.96 (1.60–5.45)	<0.001	
OS	Per one-category increase		1.72 (1.28–2.31)	<0.001	
**(B) Five-Year Survival Rates by Core-HRPF Burden Stratum**					
**Endpoint**	**HRPF Burden**	**No. at Risk at 5 Years**	**Survival (95% CI)**		
DFS	0	154	92.1% (88.6–94.6)		
DFS	1	109	83.8% (77.8–88.3)		
DFS	≥2	109	69.0% (62.8–74.5)		
OS	0	170	95.3% (92.1–97.3)		
OS	1	115	90.7% (85.4–94.2)		
OS	≥2	139	84.1% (78.6–88.3)		

Abbreviations: CI, confidence interval; Core-HRPF, core high-risk pathological feature (visceral pleural invasion [PL1/PL2], lymphovascular invasion [lymphatic or vascular], micropapillary component [>1%], solid component [any documented presence]); DFS, disease-free survival; HR, hazard ratio; OS, overall survival. Note: Core-HRPF burden is a postoperative pathological variable and is not intended as a preoperative selection criterion. The four components were weighted equally a priori for simplicity and interpretability and to avoid data-driven weighting within the same cohort; equal weighting does not imply equivalent biological or prognostic effects. The ordinal model estimates the HR per one-category increase in burden (0 → 1 → ≥2) and serves as the trend test. The surgery-by-Core-HRPF interaction was not statistically significant for DFS (*p* = 0.518). Sensitivity analyses replacing the burden count with the four individual components are provided in Table S5.

**Table 6:** Exploratory procedure-specific survival analyses in the clinicopathological cohort.

Comparison	Index-Procedure N	Index-Procedure DFS Events	Adjusted DFS HR (95% CI)	60-Month Restricted DFS HR (95% CI)	Adjusted OS HR (95% CI)
Segmentectomy vs. lobectomy	37	7	1.61 (0.74–3.49)	1.60 (0.69–3.68)	2.20 (0.87–5.56)
Wedge resection vs. lobectomy	80	16	1.82 (1.04–3.20)	0.81 (0.35–1.90)	0.76 (0.26–2.22)

Abbreviations: CI, confidence interval; DFS, disease-free survival; HR, hazard ratio; OS, overall survival. Note: N and DFS event counts refer to the index-procedure group. Procedure-specific HRs were estimated in models including lobectomy, segmentectomy, and wedge resection, with lobectomy as the reference category. Adjusted models included age, sex, maximum tumor diameter, Core-HRPF burden, and surgery year. The 60-month restricted analysis administratively censored follow-up at 60 months. Procedure-specific analyses were exploratory because of the limited numbers of patients and events, particularly after segmentectomy, and were not used to establish comparative treatment efficacy.

## Data Availability

SEER data are publicly available from the National Cancer Institute SEER program (https://seer.cancer.gov/). Clinicopathological cohort data are not publicly available due to patient privacy and institutional ethics protocols; de-identified summary data may be available from the corresponding author upon reasonable request and appropriate ethics committee approval. Statistical analysis code is available from the corresponding author upon reasonable request.

## Supplementary Materials

The following supporting information can be downloaded at: https://www.mdpi.com/article/10.3390/cancers18182941/s1, Table S1. Procedure-specific baseline characteristics and event distributions in the clinicopathological cohort (lobectomy, segmentectomy, and wedge resection). Table S2. SEER baseline characteristics, lymph-node examination distributions, and sensitivity analyses according to surgical extent. Table S3. Follow-up, proportional-hazards diagnostics, and fixed-horizon sensitivity analyses in the clinicopathological cohort, including reverse Kaplan–Meier follow-up, time-stratified Cox analyses, 60-month restricted analyses, restricted mean survival time analyses, comparable-follow-up analyses, and the exploratory 60-month landmark analysis. Table S4. Propensity-score specifications, weighting and matching summaries, and covariate-balance diagnostics. Table S5. Individual Core-HRPF component analyses and exploratory surgery-by-Core-HRPF interaction analyses. Table S6. Recurrence-event quality control, recurrence-pattern sensitivity analyses, and detailed lymph-node assessment sensitivity analyses. Figure S1. SEER procedure-specific outcome analyses for lobectomy, segmentectomy, and wedge resection. Figure S2. Propensity-score overlap and covariate-balance diagnostics in the clinicopathological cohort.
