# Supplementary material for: Real-World Outcomes After Sublobar Resection for Small Node-Negative Lung Adenocarcinoma: SEER Benchmarking and a Clinicopathological Analysis
Source: Cancers (Basel). 2026 Sep 10;18(18):2941. doi: 10.3390/cancers18182941 (PMC13604157; doi:10.3390/cancers18182941)
Supplement: Supplementary file 1 [file cancers-18-02941-s001.zip › Supplementary Figures.pdf]

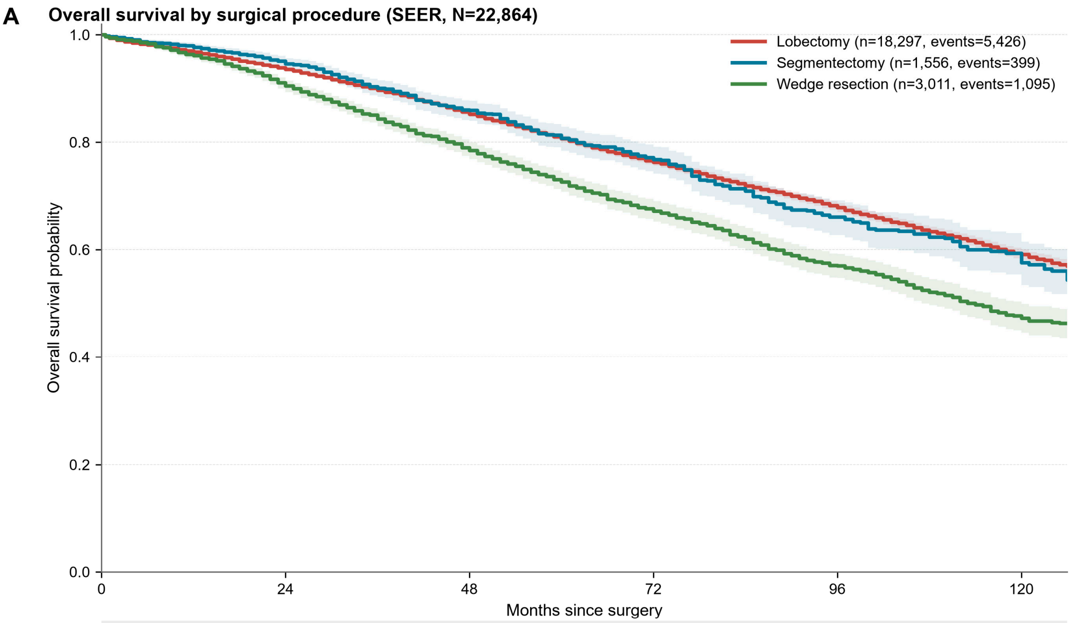

Number at risk	0	24	48	72	96	120
Lobectomy	18,297	16,920	12,709	8,705	5,448	2,952
Segmentectomy	1,556	1,454	948	565	322	166
Wedge resection	3,011	2,707	1,832	1,142	652	318

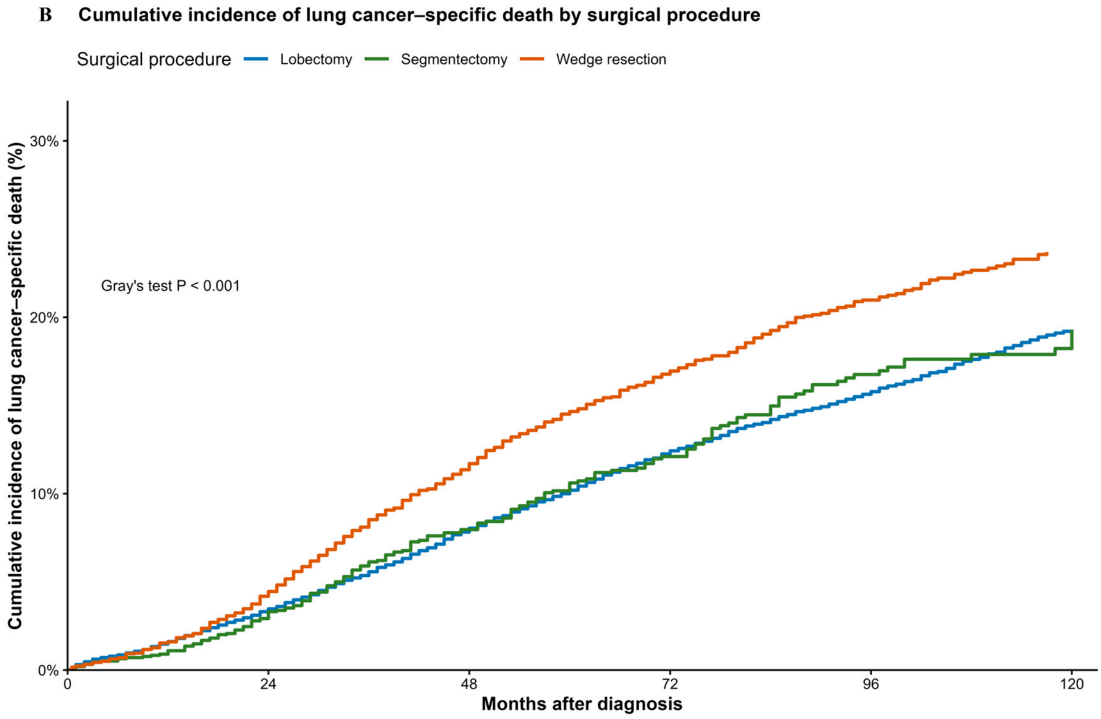

Figure S1. SEER procedure-specific outcome analyses for lobectomy, segmentectomy, and wedge resection.

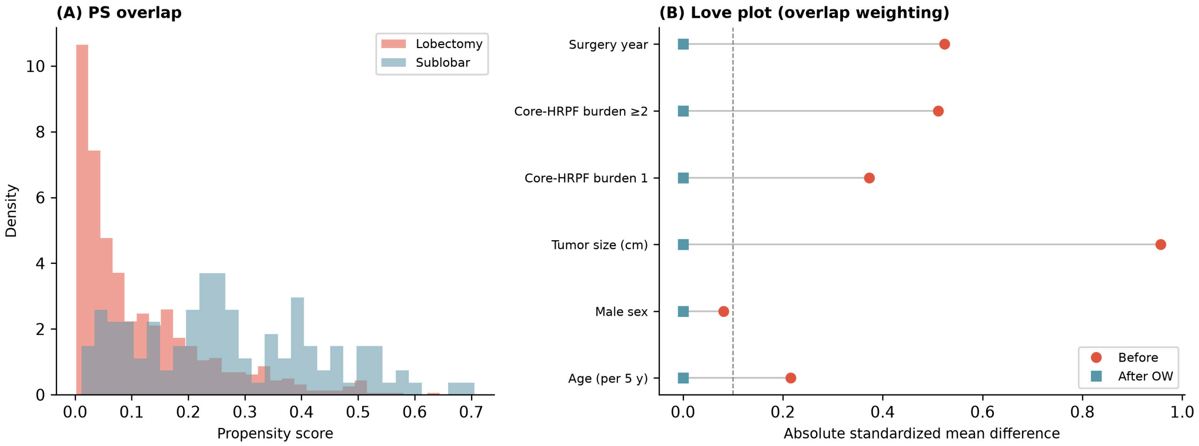

Figure S2. Propensity-score overlap and covariate-balance diagnostics in the clinicopathological cohort.
